# Options for early breast cancer follow-up in primary and secondary care - a systematic review

**DOI:** 10.1186/1471-2407-12-238

**Published:** 2012-06-13

**Authors:** Frances Taggart, Peter Donnelly, Janet Dunn

**Affiliations:** 1 Warwick Medical School Clinical Trials Unit, University of Warwick, Coventry, CV4 7AL, UK; 2 Breast Care Directorate, South Devon Healthcare NHS Foundation Trust, Lawes Bridge, Torquay, TQ2 7AA, UK

## Abstract

**Background:**

Both incidence of breast cancer and survival have increased in recent years and there is a need to review follow up strategies. This study aims to assess the evidence for benefits of follow-up in different settings for women who have had treatment for early breast cancer.

**Method:**

A systematic review to identify key criteria for follow up and then address research questions. Key criteria were: 1) Risk of second breast cancer over time - incidence compared to general population. 2) Incidence and method of detection of local recurrence and second ipsi and contra-lateral breast cancer. 3) Level 1–4 evidence of the benefits of hospital or alternative setting follow-up for survival and well-being. Data sources to identify criteria were MEDLINE, EMBASE, AMED, CINAHL, PSYCHINFO, ZETOC, Health Management Information Consortium, Science Direct. For the systematic review to address research questions searches were performed using MEDLINE (2011). Studies included were population studies using cancer registry data for incidence of new cancers, cohort studies with long term follow up for recurrence and detection of new primaries and RCTs not restricted to special populations for trials of alternative follow up and lifestyle interventions.

**Results:**

Women who have had breast cancer have an increased risk of a second primary breast cancer for at least 20 years compared to the general population. Mammographically detected local recurrences or those detected by women themselves gave better survival than those detected by clinical examination. Follow up in alternative settings to the specialist clinic is acceptable to women but trials are underpowered for survival.

**Conclusions:**

Long term support, surveillance mammography and fast access to medical treatment at point of need may be better than hospital based surveillance limited to five years but further large, randomised controlled trials are needed.

## Background

Survival from Breast Cancer has improved markedly in the last 20 years [1]. This is to be celebrated and has been attributed mainly to earlier diagnosis and new treatments to prevent recurrence [2]. Incidence of Breast Cancer however continues to increase with 47,693 new cases among women in the UK alone in 2008 [3]. The lifetime risk of breast cancer for women in the UK is now 1 in 8. This combined with increased 10 year survival to over 73% has resulted in an increase in the number of long term breast cancer survivors so that there are now over 550,000 women who have been treated for breast cancer living in the UK [3]. Survivorship after Breast Cancer and the medical, psychological and informational health needs of these patients have become increasingly recognised [4-6]. From the service provision perspective improved disease free survival reduces the burden to health services for treatment for advanced cancer but increases the burden on specialist clinics for surveillance and for surveillance mammography. There is also an increased demand for reviewing patients who refer with potential symptoms of local recurrence or new cancers which are curable if diagnosed and treated early.

In this article we have reviewed evidence for best follow up practice worldwide but because health service provision for the population varies between countries we have interpreted the evidence in the context of the UK which has a national health service (NHS) free at the point of delivery. The main objective of follow up for both patients and the NHS is the survival and well-being of patients. These two objectives can sometimes be in conflict when continued medical examinations have the potential to cause as well as relieve anxiety and to perpetuate the patient role. In this context, for example the value of the annual review and clinical examination of women who have undergone treatment for early breast cancer at the specialist hospital clinic has been called into question [7-10]. The objective of this paper is to review current evidence for women treated for early breast cancer to inform future follow up strategies.

## Methods

### Criteria for follow up

Criteria were determined by review of a broad range of literature identified from broad based searches and recent opinion articles and discussion among all three authors. MEDLINE, EMBASE, AMED, CINAHL, PSYCHINFO, ZETOC, Health Management Information Consortium, Science Direct were all used to search for these articles.

Key criteria or outcomes determined for the systematic review were as follows:

1. Risk of second breast cancer over time – incidence compared to general population (findings in Additional file 1: Table 1)

2. Incidence and method of detection of local recurrence and second ipsi and contra-lateral breast cancer (findings in Additional file 1: Table 2, Table 3)

3. Evidence of the benefits of hospital or alternative setting follow-up for survival and well-being level 1 – 4 evidence (findings in Additional file 1: Table 4, Table 5 and Table 6).

### Searches for review

**Figure 1:**
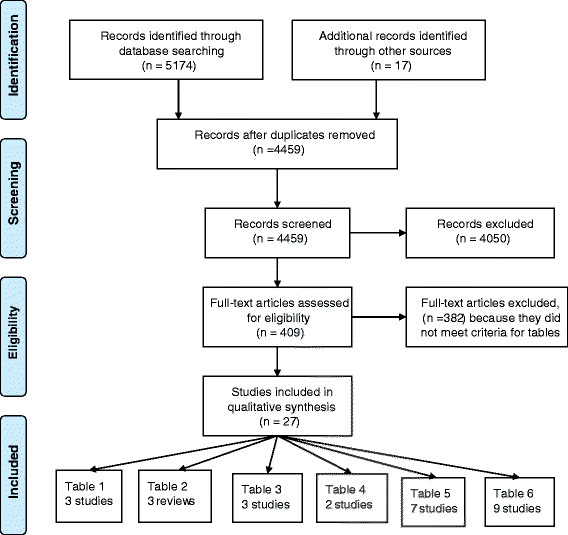
PRISMA flow chart.

Searches are reported in Additional file 2 and the Prisma flow diagrams in Figure 1. We searched Ovid MEDLINE(R) Daily Update, and Ovid MEDLINE(R) 1948-week3 2011 and Ovid MEDLINE ® In-Process and other non-Indexed Citations up to January 28 2011. For risk of contralateral cancer observational cohorts and population based studies were necessary so large population based cancer registries were used. For method of detection of recurrence related to survival, observational studies from cohorts of patients with long term follow up were necessary. For follow-up location randomised controlled trials (RCTs) of follow-up measuring survival, disease free survival, recurrence and quality of life were retrieved. All searches were from the year 2000 although one study from 1996 was included by personal communication. No language restrictions were applied. Searches were performed by FT and selection of articles independently confirmed from the title and abstracts by FT and JD.

### Patients included in the review

For all studies only articles reporting studies of women with stage 1–3 breast cancer who had no distant metastases and were in remission after surgery were reviewed. Further particulars of patients are described in the tables.

### Eligibility criteria for articles

Eligibility criteria were appropriate for the type of evidence necessary for the different follow-up criteria and are described among the inclusion criteria for each table. Manuscripts not meeting the criteria were excluded. For all tables care was taken to examine articles for possible selection bias among participants included in the studies in order to ensure generalisability of findings. For example for the reports of incidence of recurrence studies of special populations such as those of families at increased risk of cancer were excluded. Large observational cohorts of patients followed over at least 10 years with low attrition rates and population based studies using cancer registry data were suitable for estimating recurrence rates and incidence of new primaries. In order to evaluate the effect of interventions randomised controlled trials (RCTs) were the best evidence. The evidence for different follow-up locations is therefore presented in three tables according to the type and quality of evidence, firstly RCTs which include survival as an outcome, secondly RCTs which include well-being only as an outcome and thirdly observational studies and audits. Inclusion and exclusion criteria for each table are reported in Additional file 3

### Management of morbidity

For this section addressing management of morbidity the subject matter was too broad to review in a systematic way in our article but the issues were nevertheless relevant to follow up. These issues were therefore discussed in a narrative way.

## Results

### Study selection

Potential articles for inclusion in the tables were identified from the titles and abstracts from the searches in the first instance by FT and full text articles were retrieved. These and full text articles from other sources (reference lists of review articles for example) were examined by FT and JD and those meeting the inclusion criteria were included in the tables. Details of the selection of articles are shown in the PRISMA diagram in Figure 1.

Tables 1, 2, 3, 4, 5, and 6 summarise the findings of the selected studies and are presented in Additional file 1.

#### Risk of second breast cancer over time

Additional file 1: Table 1 shows the findings of studies of the risk for second breast cancer among women treated for early breast cancer. We chose to use population data for the systematic search because of the large sized populations and generalisability of findings. In Chen’s study [11] based on cancer registry data from 1970 to 1997 in Canada the incidence rate of new breast cancer was fairly constant regardless of age when the first cancer was diagnosed. Compared to the general population incidence was higher at all ages with standard incidence ratios (SIR)ranging from 16.4 (95% CI 12.25 to 21.51) in women aged under 40 to 1.28 (0.88 to 1.80) in women aged over 80 years.

Gao’s study [12] using the SEER program data showed cumulative rates for contralateral breast cancer accrued steadily over 20 years of follow up with 3% at 5 yrs, 6.1% at 10 yrs, 9.1% at 15 yrs and 12.0% at 20 years again indicating a constant incidence rate over the 20 years follow-up. Black women had a 20% increased risk compared to non Hispanic white women and women of other ethnic groups, while Hispanic and other women had a 10% decreased risk. Women with medullary cancer and women aged over 55 had a small increased risk of a contralateral breast cancer. These rates are all higher than those expected for the general population.

Soerjomatar [13] has reported a threefold increased incidence of new breast cancers among women who have had breast cancer in a population in southern Netherlands. Standardised incidence ratio (SIR) was 3.5 (3.2-3.8) among 9199 breast cancer patients diagnosed from 1972 to 2000 when compared to the population. The risk was higher among women who were premenopausal when their primary was first diagnosed. For carcinoma in situ SIR was 3.4 (2.6 to 4.3). SIR for ipsilateral and contralateral cancers was very similar, 1.9 and 2.0 respectively.

The results of other types of study confirm the findings of the studies reported above. In a systematic review of earlier studies Chen [14] reported 16 studies of cohorts of women treated for early breast cancer. Despite differences between studies in methodology and definitions of new cancers and differences in populations studied, the articles in Chen’s review consistently showed an increased incidence of contralateral cancer among women who have had a primary breast cancer when compared to the general population; incidence rate ratios were calculated in 9 studies and this ranged from 1.4 to 5.0.

#### Incidence and method of detection of local recurrence and second ipsi and contra-lateral breast cancer

The findings for this are presented in Additional file 1: Table 2 and Table 3. Local recurrence is defined as recurrence in the same breast or lymph nodes which is normally a recurrence of the same cancer as the primary. This is potentially curable with conventional therapies currently in use.

##### Incidence and method of detection of local recurrence and survival - reviews

Systematic reviews [10,15,16] of studies which described incidence and method of detection of recurrence and survival are presented in Additional file 1: Table 2. Many of the early studies included in the reviews did not distinguish between new primary cancers and local recurrence although some used histology to differentiate true recurrence. There is a consensus among the findings of these studies that hazard rates for salvageable local recurrence increases during the first two years after surgery, peaks during the third, declines until the 6^th^ year and then remains constant thereafter for stage 1 to 3 breast cancers [15,17-19]. A recent study of 650 patients treated with breast conservation from 1990 to 1997 and followed up indefinitely showed that loco-regional relapses including contralateral cancers continued to occur at a steady rate of 1.4% per year for 16 years [20]. Another recent study distinguished local recurrence from new primaries in the same breast and reported cumulative incidence of local recurrence as 5.0% (3.9-6.3) at five years, 6.5% (5.2-7.9) at ten years and 8.7% (6.2-11.6) at 15 years in a cohort of women treated with radiotherapy [21].

The earliest systematic review of method of detection of recurrences and new cancers was by Grunfeld [15] in which there was a wide range in relative percentage of recurrences detected by mammography (8-50% of cases) or physical examination (12-88%). Changes through time in frequency of use of mammography is an issue here. More recent studies tend to show a smaller proportion of cancers detected by clinical examination and a larger number by mammography. Most studies are limited by the lack of information about the mammography regimen and schedule for clinical examination.

Montgomery [10] reviewed twelve studies which measured relapse after breast conserving surgery and their method of detection. He analysed these separately in two groups one before the year 2000 and one after. Among the eight studies before 2000 46% were detected by routine clinical examination and only 15% by mammography while after 2000 40% were detected by mammography and 15% on routine clinical examination. Both Montgomery [10] and Lu [16] also looked at survival in their reviews. In Montgomery’s review relapses detected by mammography and self examination resulted in better survival at 10 years but this effect had disappeared by 15 to 20 years. Long term survival data however was only available for some studies. Lu [16] distinguished between early (mammographically detected) and late detection of recurrences in his meta-analysis and also compared mammographically detected recurrence to physical examination. In both cases mammographic detection gave better survival. This was confirmed in the recent study by Houssami [22]. Hazard ratio (HR) for asymptomatic (relative to symptomatic) detection was 0.51 (0.32-0.80) for Ipsilateral Breast Recurrence, 0.53 (0.36-0.78) for Contralateral Breast Cancer, and 0.53 (0.40-0.72) in all subjects (P < 0.0001).

Montgomery’s [19] own study showed a poorer survival for clinically detected cancers while the Dutch [23] and Hong Kong [24] studies found no difference in survival between different methods of detection but numbers involved were smaller than in the meta-analysis by Lu [16].

##### Method of detection of local recurrence and survival - articles with report of surveillance mammography published after year 2000

Since many early studies were limited by the lack of reporting of the frequency of mammography, articles with reported mammography and outpatient schedules were presented in Additional file 1: Table 3. The frequency with which recurrences were first detected by mammography was 51% in the study by Montgomery [19] in which mammography was annual throughout 10 year follow-up and 43% in the study by Yau in which mammography was annual for 5 years. Overall clinical examination was more frequent than mammography, at least every 6 months for the first five years with the exception of the Montgomery study in which clinical examination was annual after three years. The study by Lash [25] was a case control study comparing survivors with non survivors. All cause mortality rate declined with increasing number of mammograms (test for trend p = 0.007). The age- and therapy-adjusted odds ratio associating receipt of an additional mammogram compared with receipt of no mammogram was 0.77 (95% confidence interval [CI] 0.53-1.1). Despite annual mammograms for five years and frequent clinical examination 34% cancers were first detected by the patient in the study by Montgomery [26] and 9% in the study by Yau [24]. Implications of this are that self examination is still important particularly in the first six years after diagnosis.

Since recurrence from breast cancer can occur 20 years or more after treatment for the primary cancer and women who have had breast cancer are also at increased risk of developing a new cancer in the other breast, it is likely that routine surveillance mammography will be of benefit in detecting asymptomatic cancers and improving survival for an indefinite period of time. The weight of evidence supports surveillance mammography as an effective means of detecting curable recurrences and new cancers and that it improves survival. All of these observational studies of routine clinical practice showed that some cancers were still detected by clinical examination. Routine clinical examination still serves as a “safety net” for women who have not attended for mammography, do not wish to self examine, or have, for other reasons, failed to report symptoms. It is also possible that clinicians are more likely to detect recurrence in the axilla than patients who self examine. For this type of information comparison of retrospective reports of survival based on method of detection can be misleading since the method of detection will depend on the services available, the type of cancer and adequate recording of data. Slow growing cancers are more likely to be detected in the preclinical, asymptomatic stage than fast growing ones and in addition to this there is the problem of lead time bias. The studies reviewed in this section measured survival from diagnosis thus avoiding lead time bias but earlier stage detection of slow growing cancers is a source of bias that is more difficult to avoid. In future more precise characterisation of the tumour by genetic tests and surveillance with MRI may enable earlier detection than routine mammography. In a study among women at high risk of breast cancer MRI was found to be more sensitive than mammography [27]. To date MRI scanning has not been used for primary population screening because the specificity is too low but it can be effective in detecting recurrence when mammograms are difficult to read particularly after radiotherapy.

#### Evidence of the benefits of hospital or alternative setting follow-up for survival and well-being – level 1-4 evidence

There is controversy regarding the value of specialist follow up in a hospital setting as currently practised for women who have undergone treatment for early breast cancer.

##### Current practice and guidelines

The recent guidelines of the National Institute for Health and Clinical Excellence in the UK (NICE [28] guidelines 2009) recommend that asymptomatic breast cancer patients who have undergone curative treatment should have follow-up for 5 years after diagnosis. They also recommend annual surveillance mammography for 5 years after diagnosis or, for younger women, up to the age when they become eligible for the routine population screening programme. A survey of specialist breast care practitioners in the UK (before the most recent guidelines) by Donnelly [29] demonstrated that 92% were discharging patients according to a locally agreed protocol. Decisions about follow-up were made based on risk of recurrence and prescribing of aromatase inhibitors or tamoxifen. Clinicians felt that the follow-up clinic visit was mainly aimed at managing side effects of medication in order to maximise compliance, treating treatment sequelae, detecting recurrence and new cancers and identifying psychological problems.

ASCO [30,31] recommend a careful history and physical examination every 3–6 months for the first three years, every 6–12 months for the 4^th^ and 5^th^ year and annually thereafter. They also recommend that in addition to the physical examination and history physicians should counsel patients about symptoms of recurrence and about breast self-examination. Women at high risk for familial breast cancer syndromes should be referred for genetic counselling. Pelvic examinations are also recommended for all women particularly patients taking Tamoxifen (who are at increased risk of endometrial cancer) and re-referral for oncology assessment for all women receiving adjuvant endocrine therapy.

##### RCTs of breast cancer follow-up in hospital or alternative settings which include recurrence or survival as outcomes - level 1 evidence

By far the greatest limitation to the validity of these studies (Additional file 1: Table 4) is the significant number of patients excluded by the medical staff or breast care nurses because they considered them unsuitable. Reasons for this were not always given but it is likely that reasons are related to clinical issues such as post-surgery problems or perceived risk of relapse or anxiety on the part of the patient.

The findings need to be interpreted in this context. Grunfeld’s two studies of hospital follow up compared to follow-up in General Practice in the UK [32] and in Canada [33], respectively, provide information on survival and well-being (18 month and median of 3.5 years follow up respectively) and show no differences in overall survival together with general satisfaction of patients and no difference in well-being. Grunfeld’s Canadian study remains the largest study to date reporting recurrence and survival endpoints based on an analysis of 968 patients. The studies were, however underpowered and follow up was too short to evaluate the impact on survival. Similarly reports of nurse led follow-up in the UK such as Beaver’s study [34] report high levels of satisfaction but have small numbers of patients. Beaver’s study had a follow-up period of five years so that long term survival could not be measured but it was possible to measure time to confirmation of recurrence in hospital and this was not different in the two groups. In conclusion, data on survival is inadequate and the effects of alternative follow-up on survival remain unknown.

##### RCTs of breast cancer follow-up in hospital or alternative settings with acceptability, well-being, access to medical care as outcomes - level 1 evidence

There is a consensus among the RCTs of nurse led or General Practitioner (GP) led alternative follow-up that patient satisfaction and anxiety is similar or better in alternative follow-up (Additional file 1: Table 5). The findings indicate no difference in health related quality of life [35-39]. Patient satisfaction was better in the GP group in Grunfeld’s study [40]. There was no evidence for increased use of services in GP follow up in one study [41]. In the RCT by Sheppard [42] the majority of patients who had a recurrence in both the point of need access group and the 6 monthly review group were admitted via an emergency route. The short symptom history indicated that it was unlikely that the recurrences would have been detected at a routine visit.

There is however a paucity of evidence for evaluating different locations for follow-up. New studies with limited findings as yet are in progress; a four arm RCT in the Netherlands comparing hospital and nurse led follow-up has shown evidence for acceptability by patients of alternative nurse led follow up but it is too soon yet for survival evidence [43-45] there is also a shared care study of GP follow-up in France at the Institut Curie [46].

##### Breast cancer follow-up in alternative settings with acceptability, well-being, access to medical care as outcomes - observational studies or audits - level 2–4 evidence

Evidence from observational studies and audits to evaluate alternative follow-up compared to hospital follow-up for outcomes such as well-being and satisfaction and in some cases survival is shown in Additional file 1: Table 6.

There is also no evidence of increased use of normal GP services or increased numbers of tests in the alternative groups where this was investigated. Koinberg [35] in Sweden has compared costs of routine follow-up by a physician with nurse led follow up on demand for five years and found the nurse led follow-up to be less expensive. Patient satisfaction was high and anxiety low in both groups.

Evidence regarding the psychological effect of the annual visit itself on the patient is conflicting with some studies showing that patients are reassured by the visit and others reporting that it generates anxiety [34]. Ganz [4] reports that women often report that their fear of recurrence increases after active treatment is withdrawn and they miss the reassurance that ready access to the health care system can provide. Long term shared care protocols which incorporate continuity of care for patients could address many of the medical and psychosocial needs of patients.

Lash [47] in a USA study of a cohort of stage 1–2 breast cancer patients in five hospitals in Boston compared patients who received USA guideline surveillance consisting of history taking, clinical examination and mammography with those who did not. Mortality and cancer related anxiety were higher among patients who did not receive surveillance. This persisted after controlling for a number of confounding factors such as age, primary therapy, cardiopulmonary co-morbidity index, education and other social covariates. This was an observational study and so the findings may have been affected by social factors for which it was not possible to control. Chapman [48] audited a scheme of patient led follow-up (PLFU) for low risk patients in Cambridge and found that the scheme was universally well received by patients and did not significantly increase GP workload. The PLFU includes an education session for the patient and an information pack at their follow up discharge interview and there is also educational material for the patients’ GPs. 126 (97%) patients had a clear idea of how to contact the breast unit, and only 5 of 130 patients (4%) required a breast clinic appointment. Only 10 of 277 GP respondents (3.6%) referred a patient on PLFU back to the breast unit during the study period. In South Wales a radiographer led follow up has been piloted [49]. Under this scheme the patients have a one stop service whereby the radiographer goes through a protocol set of questions and physical examination at the time of the mammographic surveillance. If there are any problems there is a fast track referral service to the breast cancer unit at the hospital.

In Scotland Montgomery [50] assessed the acceptability of automated telephone follow up in a feasibility pilot study of 110 women who attended a routine follow up clinic between May to August 2006. They found that 71% of the patients found the system easy to use but only 65% liked it and were happy to use it as their sole means of follow-up. All of the UK schemes also incorporate telephone access to a specialist breast cancer nurse and are underpinned by surveillance mammography.

Jiwa [51] in a study of follow up in general practice found anxiety and depression presented relatively soon and were often enduring whereas concomitant medical problems also presented later.

Murray [52] reported a service user designed framework for proactive care for people with cancer in five General Practices in Scotland. Innovations included an intranet based register, meetings and information sheets. Patients, family carers and professionals suggested that the framework helped achieve continuity of care and improved support and information for all.

Vanhuyse [53] reported a Canadian programme of planned discharge to family physician including patient and also family physician information packages. They report reasons for not transferring. However patients transferred were still seeing radiologists and surgeons.

In addition to the intervention for alternative follow up patients in the UK normally have access to a specialist breast care nurse for an indefinite period of time after their operation and this may be helping to reassure women discharged from hospital follow-up in the UK studies. The non-randomised evidence of cohorts or observational studies contributes to the generalisability of the findings of the RCTs since they are based on a broader base of patients.

### Management of morbidity

#### Evaluation of symptom oriented detection of distant metastases with clinical examination versus more intensive investigations

It is clear that early detection of local recurrence and early detection of new cancers can improve survival. However ASCO (2006) [31], NICE (2009) [28], BASO(2009) [54] and ESMO(2008,2009) [55,56] guidelines are unanimous in advising against routine search for distant metastases. There is no advantage in early diagnosis of distant metastases since there is no evidence that early treatment is more effective. Evidence for this mainly comes from a Cochrane review in which the use of intensive surveillance using bone scans and blood tests for tumour markers in order to search for distant metastases was evaluated [57]. Within the review two RCTs involving 2563 women compared usual follow up of clinic visits and mammography with more intensive investigations [58,59]. The findings were that routine screening for metastases using MRI scans and blood tests for tumour markers did not improve survival (hazard ratio 0.96, 95% confidence interval 0.80 to 1.15) or disease-free survival (hazard ratio 0.84, 95% confidence interval 0.71 to 1.00) or quality of life. Reporting symptoms and starting treatment when they occur, surveillance mammography and clinical examination were as effective. There was still no difference in survival after 10 year followup in the Roselli del Turco study [59]

#### Adherence to endocrine therapy and management of side effects

These treatments are long term preventive therapies and adherence to endocrine therapy has been cited as one of the main reasons for hospital led follow-up by hospital consultants in the UK [60]. In a Dutch study [61] adherence to hormone therapy was better than in the UK and the authors presumed this was due to five years follow up as opposed to three in the UK. Chemotherapy and treatment with Herceptin occurs within the hospital setting prior to discharge to routine surveillance. However hormone therapy, either tamoxifen or aromatase inhibitors, are being used for increasing periods of time to reduce the risk of local recurrence and metastatic disease. Adjuvant hormonal or chemotherapy has also been found to considerably reduce the risk of a new contralateral breast cancer primary [62-64]. Numerous trials have shown the effectiveness of Tamoxifen and aromatase inhibitors in increasing survival in women with ER + ve breast cancer and treatment with tamoxifen and aromatase inhibitors is now standard practice. Ongoing trials with new drugs will indicate the optimum length and sequence of follow-up treatment with different drug combinations. Chlebowski [65] performed a systematic review of 9 trials of adherence to endocrine therapy in clinical settings. Findings were that in adjuvant breast cancer clinical trials with greater or equal to 4 years follow-up, hormonal therapy (tamoxifen or aromatase inhibitors) was prematurely discontinued by about 23-28% of the study participants. Adherence to aromatase inhibitors did not differ from adherence to tamoxifen in this setting. In breast cancer prevention trials, tamoxifen was prematurely discontinued by 20-46% of the participants. In clinical practice settings, only 2 reports addressed longer-duration (>4 years) adherence to adjuvant tamoxifen use. In these, tamoxifen was prematurely discontinued by 30-50% of the patients. Poor tolerance of treatment adverse effects was reported in older breast cancer survivors and this predicted mortality at 7 years follow up in a further recent study by Clough-Gorr [66].

In the light of the effectiveness of these drugs in reducing breast cancer mortality compliance is a problem which should be addressed in follow up. Reasons for non-compliance are not clear but side effects are likely to be one cause. These include the effects of long term anti-oestrogen therapy such as osteoporosis and possible effects on lipid metabolism and cardiovascular risk which should be monitored in all patients. There is a need for further research in this area.

#### Interventions to improve well-being among women treated for early breast cancer

Long term symptoms and after effects of treatment that women who have had breast cancer can encounter are well known. Ganz [4-6,67,68] has reported these symptoms extensively in several publications including a large survey of breast cancer survivors 5 to 10 years after diagnosis. She has recommended a shared care follow-up plan including a record of treatment to be held by the patient. Many side effects associated with adjuvant endocrine therapy can continue for 5–10 years and patients are at increased risk of thromboembolic disease, uterine cancer and possibly cerebrovascular events. Patients on aromatase inhibitors also are at increased risk of osteoporosis and fractures. Patients also have long term after effects of systemic therapy. These include fatigue, ovarian failure, and menopausal symptoms, neuropathy, cognitive dysfunction, weight gain, psychological distress and sexual dysfunction. Late complications include an increased risk of leukemia and an increased risk of cardiac dysfunction due to anthracyclines. There are also long term side effects following surgery and radiation therapy including numbness, weakness and arm swelling. Breast pain may result from radiation therapy in 1% of patients. Anxiety and depression are also common. Lebel [69,70] followed survivors in a study of stressors at 5 time points up to 6 yrs post diagnosis. Cancer concerns were rated as not especially stressful, with the exception of fear of the future which was the most stressful of the four concerns on all measurement occasions. Physical limitations and pain were reported to induce equivalent levels of stress and their intensities decreased over time. Patients in the UK may receive medical treatment for these symptoms routinely through their General Practitioner. The Cochrane review by Cruickshank [71] revealed that psychosocial nursing interventions around diagnosis and early treatment could affect some components of quality of life, such as anxiety and early recognition of depressive symptoms. However, their impact on social and functional aspects of the disease later on was less clear. Physical exercise seems to be the most effective strategy to combat fatigue and to improve mood [72-79]. Other interventions involving information and psychosocial support had variable levels of success but most report benefits [38,71,80-85]. In one trial [86] yoga was found to be effective for many well-being outcome measures. Information and support were most effective in the first three months after diagnosis. The study by Sandgren [38] was a nurse led telephone intervention incorporating education and this showed one small effect for perceived stress favouring health education. The psychological intervention by Andersen [87] reported that survival at 11 years was improved (recurrence hazards ratio [HR] of 0.55; P = 0.034) and death from breast cancer (HR of 0.44; P = 0.016) in addition to improved well-being and compliance with medication.

## Discussion

This study has highlighted evidence for follow up strategies that are likely to improve survival and well-being of women treated for early breast cancer. Follow up should encompass early detection of new cancers as well as recurrence and maximise adherence to preventive endocrine therapy in those patients with oestrogen or progesterone receptor positive cancers. Evidence that women with a history of breast cancer are at increased risk of a second breast cancer primary was confirmed in large populations with different health care systems. Long term studies indicate that this increased risk continues at a constant rate for 20 years or more.

There is evidence from the studies we reviewed that mammographically detected recurrences are also detected at an earlier stage and results in better survival than clinically detected ones and that local recurrence may occur many years after treatment. The finding that a regimen of surveillance mammography offers a survival benefit among women treated for primary breast cancer when compared with a surveillance regimen that does not include surveillance mammography was also reported in a recent Health Technology Assessment incorporating a systematic review [88]. However due to the limited availability of data the studies reviewed were not randomised controlled trials and no conclusions could be drawn about the optimum frequency or duration of mammography after surgery. It is also unclear as to what age mammography should be continued. A pragmatic approach is to only undertake investigations if the findings would influence clinical decisions. In women over the age of 80 many would elect not to have surgery because of the higher risks in this age group. If there is already considerable comorbidity adding preventive medication with likely side effects and drug interactions may not be advisable. There is the potential for harm due to over-surveillance in this case. In the same way a search for distant metastases in healthy women treated for early breast cancer is not advised and this is generally accepted among breast cancer specialists.

Whilst much of what we know about the benefits and side effects of breast cancer treatment have been learnt from long term follow up of cohorts of patients in the specialist clinic its contribution to improved outcomes remains unclear. Detection of local recurrence and contralateral cancers occurs more often by patients or by surveillance mammography than by routine clinical examination and hospital based follow up does not meet patients’ needs for psychosocial support. The increasing numbers of breast cancer survivors put pressure on services that may be better directed to patients who are ill. Recent debate [89] regarding the possibility of “over diagnosis” of breast cancer through screening [90] has highlighted the pivotal role of long term tracking of cohorts of patients in evaluating the prognosis of small cancers. It may be possible in the future to identify genetic profiles and patient characteristics which predict a very low risk. This need not be a reason for hospital led follow-up; computerised tracking and linking of primary care records would enable this essential data to be recorded for research purposes.

In trials of alternative follow-up after one year a significant proportion of patients were retained in hospital follow-up by oncologists [32,33]. It is likely that patients requiring mastectomies and chemotherapy may not have completed treatment by one year after diagnosis and patients may also be retained because of anxiety. Early discharge to alternative follow up at one year post diagnosis is likely to be suitable only for low risk patients who have had conservative surgery with no complications or need for reconstruction.

Addressing emotional and physical concerns are important parts of survivorship which should be incorporated into any follow-up plan regardless of location. Ganz recommends a self held care plan. In the UK the universal population based primary care system includes general practitioners, practice nurses, district nurses, health visitors and community psychiatric services which often work from the same health centres. Patient held care plans are successfully used for a variety of chronic conditions as a supplement to the NHS organisational structures and as an aid to communication.

Generally women reported high satisfaction with alternative follow-up regimes. These studies did not report any consideration of age in the design or interpretation of the trials or details of how alternative follow-up was presented to the women and whether survival was discussed. It is likely that when survival and well-being are in conflict, such as in making decisions about stressful tests or preventive treatment which has side effects, patients may make different choices [66] so that some inequalities will not depend on service availability. More research is needed into how well patients understand risk, how much they are prepared to allow their treating doctors to make medical decisions on their behalf and the social differences and circumstances associated with these choices.

A major issue in follow-up is the management of preventive hormone therapy. 75% breast cancers are hormone dependent and thus susceptible to hormone therapy. In a survey of breast cancer specialists in the UK the management of this therapy was highlighted as the most important aim of follow-up [29]. Preventive treatment and the management of chronic disease is typically the premise of General Practice not the specialist unit at the hospital. In the light of new preventive treatments available and the need to monitor long term side effects such as osteoporosis, it is likely that the majority of this care should be transferred to General Practice where informational needs for local support networks and other services could also be more easily met.

A strength of our study is that it is a synthesis of evidence and encompasses a range of important criteria for breast cancer follow-up both for the survival and well-being of patients and health service provision. We have incorporated other more specialised reviews among our evidence. A limitation is that we have not included an analysis of cost with the exception of one study where the evidence was from a randomised controlled trial[35]. We chose not to review other studies based on theoretical models using assumptions for input data.

## Conclusions

Long-term support, surveillance mammography and fast access to medical treatment at point of need may be better than hospital based surveillance limited to five years. Women who have had breast cancer are at increased risk of a second primary in the long term and this is particularly important for younger women. The frequency with which local recurrence is detected by patients between routine surveillance mammography indicates that breast self-examination may be important for this group and a risk adjusted surveillance strategy may be helpful. Surveillance mammography and transfer to management in General Practice or a nurse or radiographer led service operating from the hospital are acceptable to patients but adequate data on survival is lacking. Evidence for optimal frequency and duration of surveillance mammography is inadequate. Further studies with long term outcomes are needed to establish the safety and effectiveness of novel alternative options relevant to patient cohorts stratified for age, tumour biology and treatment type.

## Competing interests

The authors declare that they have no competing interests.

## Authors’ contributions

FT identified criteria, did searches, extracted data, selected articles and wrote text. JD identified criteria, selected articles, contributed to and reviewed text. PD identified criteria, reviewed selected articles, contributed to and reviewed text. All authors read and approved the final manuscript.

## Name of guarantor

Guarantor: Professor Janet A Dunn

## Ethics approval

Not applicable

## Funding

A NIHR senior investigator award for Professor Janet Dunn funded this research. The researchers were independent of the funders.

## Pre-publication history

The pre-publication history for this paper can be accessed here:

http://www.biomedcentral.com/1471-2407/12/238/prepub

## Supplementary Material

Additional file 1 **Tables selected articles [**10,12-16,19,24,25,32-34,36,37,40-43,45,48-53**].** Click here for file

Additional file 2 Searches. Click here for file

Additional file 3 Inclusion criteria. Click here for file
