# Supplementary material for: Options for early breast cancer follow-up in primary and secondary care - a systematic review
Source: BMC Cancer. 2012 Jun 13;12:238. doi: 10.1186/1471-2407-12-238 (PMC3502561; doi:10.1186/1471-2407-12-238)
Supplement: Additional file 1 — Tables selected articles [10,12-16,19,24,25,32-34,36,37,40-43,45,48-53]. [file 1471-2407-12-238-S1.pdf]

ADDITIONAL FILE 1 - Tables

Table 1 Risk of second breast cancer over time – population studies

Author	Year	Country	Type of Study	Population	Sample size	Findings
Gao[12]	2003	USA	Population based longitudinal study using SEER database of breast cancer (1973-1996)	11 population based cancer registries representing 14% of population of USA	134,501 women with localised primary unilateral invasive breast cancer (115,606) or with DCIS (18,895)	The 10 and 20 year actuarial rate for cancer in the other breast or contralateral breast cancer (CBC) was 6.1% and 12% respectively. The cumulative rates for CBC accrued at a constant rate over 20 years of follow up. Radiotherapy was associated with a small increased risk among women aged <45 yrs (RR=1.32 p=0.01)
Chen[14]	2001	Canada	Population based longitudinal study using Manitoba Cancer Registry (1970 - 1997)	Population based cancer registry for the province of Manitoba, Canada	14,220 women with primary invasive breast cancer (no DCIS).	Women who had breast cancer were more likely to get a second primary than women of similar age in the Manitoba general female population. Standardised incidence ratio (SIR) was 16.40 (12.25 to 21.51)* for women aged <40yrs, 3.99 (3.32 to 4.74)* for 40-49yrs. SIR then declined in 10 year age groups to 1.28 (0.88 to 1.80)* in women aged over 80.
Soerjomataram [13]	2005	Netherlands	Population based longitudinal study using Eindhoven Cancer Registry data (1972 - 2000)	Population based cancer registry for Eindhoven covering 2.4 million inhabitants.	9919 women with primary breast cancer. Mean follow-up was 6.6 years (no DCIS).	Overall SIR for a second breast cancer primary compared to the general population was 3.5 (3.2 to 3.8)*. For premenopausal women SIR was 6.3 and for post menopausal women SIR was 2.6.

*95% confidence intervals

Table 2 Incidence and method of detection of local recurrence and survival – reviews

Author	Year	Type of Study	Patient group	Findings
Grunfeld[15]	2002	Systematic Review of 15 prospective observational studies from 1990 to 1999	Mostly breast conservation treatment. From 102 to 7000 patients.	Ipsilateral recurrence detected by mammography alone ranged from 8% to 50% and by physical examination ranged from 12% to 88%. In two studies recurrences discovered by mammography were smaller and had less invasive characteristics. In one study recurrence detected by mammography resulted in better 5 year survival. In four studies where incidence of ipsilateral recurrence and contralateral cancer incidence were both reported the incidence of contralateral cancer was higher.
Montgomery[10]	2007	Systematic Review of 8 studies before 2000 and 4 between 2001 and 2007	12 studies involving 2921 early breast cancer patients	In studies published before 2000, 15% of relapse was mammographically detected with 46% detected by routine clinical examination. In those published after 2000, 40% were mammographically detected with 15% detected on routine clinical examination. Patients with ipsilateral breast relapse detected clinically appeared to do less well than those with relapse detected by self-examination or mammography in terms of survival at 10 years but this difference disappeared by 15-20 years and overall there was no significant difference in survival between the three groups. Long term follow-up not available for all studies.
Lu[16]	2008	Systematic review and Meta-analysis of method of detection and survival	2263 patients from 13 observational studies	Detection of breast cancer recurrences by routine mammography in asymptomatic patients gave a significantly better survival compared to recurrences detected by breast self examination or symptoms HR: 1.68 (95% CI: 1.48–1.91). Survival was better when the recurrence is found by mammography instead of physical examination (HR: 2.44 (95% CI: 1.78–3.35). The findings were not accounted for by lead time bias.

Table 3 Method of detection of local recurrence and survival – cohort studies with report of surveillance mammography published after year 2000

Author	Year	Country	Type of Study	Patient group	Outpatient follow-up schedule	Mammogram frequency	Age	Outcome measure	Findings
Montgomery[19]	2007	UK	Prospective observational study. Patients followed 10yrs	1312 patients treated with breast conserving treatment between 1991 and 1998	3–4 monthly visits for the first 2 years, 6 monthly for 3 years then annual visits until the tenth anniversary.	Annual bilateral mammography throughout	24-91yrs mean 56	Pattern of relapse and detection<br><br>5 and 10 yr survival	15 (14%) detected by clinical examination, 37 (34%) by patients and 56 (51%) by mammography. Patients with mammographically detected or self detected ipsilateral breast relapse had longer survival from original diagnosis (P=0.0002) and from recurrence (P=0.0014) compared with those detected by clinical examination.
Yau[24]	2008	Hong Kong	Prospective, observational study of patients followed for 5.9 yrs	511 (495 Chinese) women with invasive and non invasive breast cancer	clinical examination every 2-3 months for 2 yrs then every 4-6 months up to 5 yrs	Annual mammography for 5 yrs then every 1-2 yrs for further 5 yrs	25-90 median 46	Local recurrence rate in same breast and method of discovery	23 (4.5%) patients had local ipsilateral recurrence. 10 (43%) were detected by mammography, 3 (13%) by ultrasound (negative mammograms) and 6 (26%) by clinical examination and 2 (9%) were detected by the patient. No differences in survival between detection methods were found.
Lash[25]	2006	USA	Case control analysis of prospective cohort survivors vs dead by no mammograms	334 65 yr old women with stage 1-3A breast cancer	Clinical examination not relevant to this case control study design which compared number of mammograms in survivors vs dead.	Number of surveillance mammograms from first day of surveillance until 6 months before date of death recorded for cases and for 4 matched controls for the same follow up time.	age 65 and over	All cause mortality	All cause mortality rate declined with increasing number of mammograms. Test for trend p=0.007. The age- and therapy-adjusted odds ratio associating receipt of an additional mammogram, compared with receipt of no mammogram, was 0.77 (95% confidence interval [CI] 0.53-1.1).

Table 4 Randomised controlled trials of breast cancer follow-up in hospital or alternative settings which include recurrence or survival as outcome – level 1 evidence

Author	*Year*	*Country*	*Study groups*	*Sample size*	*Outcome measure*	*Findings*
Beaver[37]	2009	UK	Telephone follow-up by breast care nurses compared to doctors	374	Time to detection of recurrence, psychological morbidity (Spielberger state anxiety inventory, General Health Questionnaire - GHQ12), information needs, participants satisfaction.	Patients followed for 24 months. Only 17 participants had a confirmed recurrence 6 in hospital group and 11 in the telephone group and there was no significant difference between groups in time to confirmation (60 vs 39 days in hospital and telephone group respectively). No significant differences were found in other outcome measures except satisfaction with information received which was higher in the telephone group.
Grunfeld[35]	2006	Canada	Follow-up in the cancer center (CC) or family practitioner (FP)	968	Primary outcome was the recurrence-related serious clinical events (SCEs). The secondary outcome was health-related quality of life (HRQL)	In the FP group, there were 54 recurrences (11.2%) and 29 deaths (6.0%). In the CC group, there were 64 recurrences (13.2%) and 30 deaths (6.2%). In the FP group, 17 patients (3.5%) compared with 18 patients (3.7%) in the CC group experienced an SCE (0.19% difference; 95% CI, 2.26% to 2.65%). No statistically significant differences were detected between groups on any of the HRQL questionnaires

Table 5 Randomised controlled trials of breast cancer follow-up in hospital or alternative settings with acceptability, well-being, access to medical care as outcomes – level 1 evidence

Author	*Year*	*Country*	*Groups compared*	*Sample size*	*Outcome measure*	*Findings*
Grunfeld[36]	1996	UK	Specialist at multidisciplinary breast clinic compared to General Practitioner	296	Time from first symptoms to diagnosis of recurrence; quality of life by SF-36 and EORTC, and HADS questionnaires.	Most recurrences (18/26, 69%) presented between routine visits, and almost half (7/16, 44%) of the recurrences in the hospital group presented first to general practice. The median time to hospital confirmation of recurrence was 21 days in the hospital group (range 1-376 days) and 22 days in the general practice group (range 4-64). There were no significant differences between groups in the change in mean scores in any of the measures of quality of life or anxiety and depression from baseline.
Gulliford[44]	1997	UK	Conventional schedule of clinic visits or visits only after mammography	196	Acceptability, interim use of telephone and general practitioner, satisfaction.	After randomisation more patients expressed a desire to reduce further, rather than increase, the frequency of follow up. Patients undergoing less frequent review did not increase their use of general practitioner or telephone (hotline) services
Koinberg[39	2004	Sweden	Physician group (PG) or on demand by a specialist nurse (NG)	264	Well-being, satisfaction, access to medical care and medical safety.	Levels of anxiety and depression were generally low and levels of patient satisfaction high. There were no differences between the groups concerning time to recurrence or death.
Brown[40]	2002	UK	Standard clinic follow up and patient initiated follow up	61	Quality of life, psychological morbidity, satisfaction with follow up, contact with healthcare professionals.	There were no major differences in quality of life and psychological morbidity between the groups although more women in the standard clinic group reported reassurance and being checked as advantages whereas more women in the patient initiated follow up group reported convenience as an advantage.
Grunfeld[43]	1999	UK	Standard clinic follow up or care by their GP	296	Satisfaction and change in satisfaction by questionnaire administered three times in 18 months	GP group selected responses indicating greater satisfaction than hospital group and there was an increase in satisfaction in the GP group but not in the hospital group.

Sheppard[45]	2009	UK	Standard 6 monthly review for 2 yrs post diagnosis or point of need access	237	Psychological morbidity using General health questionnaire (GHQ12) at baseline, 9 and 18 months quality of life using the FACT-B plus endocrine subscale, fear and isolation.	There were five recurrences in the point of need access group and four in the control group. The majority of patients with recurrence in both groups were admitted via an emergency route. The presentation of recurrences and short symptom history in both groups demonstrate that the recurrences observed were unlikely to have been detected at a routine visit. Patients utilised point of need access effectively with no excessive use of access via the specialist nurse and there was no difference in GHQ. Also no difference in fear or isolation after 18 month follow up Evidence that a flexible service determined by patient initiative has no disadvantages (after 18 months)
Kimman[46]	2010	Netherl ands	3-monthly outpatient visits or nurse led telephone follow up or 3 monthly outpatient visits with an educational group programme	320	Health related quality of life (HRQoL) measured by EORTC QLQ-C30 questionnaire to for cancer patients. Also role and emotional functioning and feelings of control and anxiety.	No significant difference in HRQoL between nurse-led telephone and hospital follow-up. At 12 months 95% CI for difference was -1.93 to -4.64 p=0.42. No significant difference between nurse-led telephone and hospital follow-up for any of the other outcome measures.

Table 6 Observational studies or audits of breast cancer follow-up in hospital or alternative settings which include acceptability, well-being or access to medical care as outcomes. – Level 2-4 Evidence of survival or well-being

Author	*Year*	*Country*	*Type of Study*	*Sample size*	*Outcome measure*	*Findings*
Lash[25]	2005	USA	Case-control comparison of guideline surveillance vs. no surveillance in stage I - II breast cancer cohort	303	Mortality. Cancer-related worries from four questions rated on 5 point scale, SF-36 and covariates assessed by telephone interview at 3 and 21 months after diagnosis and annually thereafter.	After median follow-up 7.4 years women who had guideline surveillance including physical examination were significantly less likely to have died. After adjustment for age, primary therapy, cardiopulmonary co-morbidity index, education and other social covariates the odds ratio associating guideline surveillance in the preceding year with all-cause mortality was 0.66 (95% CI = 0.51-0.86). The adjusted odds ratio associating continuous guideline surveillance with an increase in cancer-related worries was 0.37 (95% CI = 0.14-0.99).
Chapman[51]	2009	UK	Audit of patient satisfaction with Cambridge Patient Led Follow Up for low risk breast cancer patients.	217	Patient satisfaction	All 106 respondents (100%) were satisfied with the process to contact the breast unit. The introduction of a Patient Led Follow Up protocol (PLFU) for low risk breast cancer patients was well received by patients. Only 10 of 277 GP respondents (3.6%) referred a patient on PLFU back to the breast unit during the study period. PLFU has been well received by patients following breast cancer treatment with little increase in GP workload.
Montgomery53]	2009	UK	Trial of acceptability	110	Semi-structured interview to asses acceptability of automated telephone questionnaire to replace annual follow up visit (all patients had annual mammogram)	Seventy-five patients (71%) completed follow-up using the new automated system 1 year later. Seventy-one of the 75 patients found the system easy to use. Forty-nine of the 75 (65.33%) liked the system and were happy to use it as their sole method of follow-up. A further 12% were happy to use it as part of their follow-up. In 10.66% of participants concerns were raised which led to clinic attendance.
Jiwa54]	2006	Australia	Survey and focus groups	702 patient years	Survey of most common problems reported in General Practice after breast cancer	The most frequently recorded problems were anxiety, unrelated medical problems and joint pain. Anxiety and depression tend to present relatively soon and are often enduring whereas concomitant medical problems also present later. Health care professionals considered patients difficult to manage because symptoms of recurrence require

						investigation for absolute reassurance of the symptomatic patient.
Murray[55]	2008	UK	Framework for care for people with cancer in five General Practices	18 patients and carers and 49 professionals	Describe local innovations for proactive care, information and support and report perceptions of patients, carers and professionals	Local innovations included an intranet-based register, information sheets and regular multidisciplinary meetings. Patients, family carers and professionals suggested that the framework had helped achieve continuity of care, teamwork, proactive care and improved support and information for patients and carers.
Vanhuyse[56]	2007	Canada	Discharge from cancer centre to family physician.	193	83 patients discharged 110 deemed unsuitable	Reasons for not transferring: clinical trial enrolment (50.9%), ongoing endocrine treatment (31.8%), new symptoms (6.3%), and patient refusal (0.9%). Patients transferred to family physicain were still seeing radiologists and surgeons.
Vaile[52]	2006	UK	Description of Velindre model: Radiographer led follow-up service.	6500 attendees per year for 3yrs	Describes first year of running system with 949 patients.	Radiographer trained in symptoms of breast cancer recurrence and administration of a questionnaire for patients. Referral to breast cancer nurse or oncologist was according to set protocols. 949 patients were taken out of outpatient clinics, with the result that those clinics were less fraught. This made extra time available to deal with new patients or those with ongoing medical problems.
Kimman[48]	2009	Netherlands	RCT Multicentre trial of 4 follow up strategies .	192	Subscale global health of the disease-specific HRQoL measure and EORTC QLQ-C30 The two HRQoL measures were completed two weeks and one year after finalizing curative treatment.	This study reports responsiveness of the EQ-5D in breast cancer patients. Four strategies compared 1) standard follow-up; 2) nurse-led telephone follow-up; 3) arm 1 with the educational group programme; 4) arm 2 with the educational group programme. Data is collected at baseline and 3, 6, 12 and 18 months after treatment. The EQ-5D was able to capture both improvements and deteriorations in HRQoL. Final findings of trial not yet available.
Koinberg[38]	2009	Sweden	As above	264	As in previous paper (see table 5) and cost per person year of follow-up	The cost per person year of follow-up differed between the groups, with 630 euro per person year in PG compared to 495 euro per person year in NG. The main difference in cost between the groups was explained by the numbers of visits to the physician in the respective study arms. There were 21% more primary contacts in PG than NG.
