# Supplementary material for: Options for early breast cancer follow-up in primary and secondary care - a systematic review
Source: BMC Cancer. 2012 Jun 13;12:238. doi: 10.1186/1471-2407-12-238 (PMC3502561; doi:10.1186/1471-2407-12-238)
Supplement: Additional file 2 — Searches. [file 1471-2407-12-238-S2.pdf]

ADDITIONAL FILE 2 Searches

Follow up trial search

Database: Ovid MEDLINE(R) <1948 to January Week 3 2011>, Ovid MEDLINE(R) Daily Update <January 28, 2011>, Ovid MEDLINE(R) In-Process & Other Non-Indexed Citations <January 28, 2011>

Search Strategy:

----

1 (follow up or follow-up or surveillance).mp. [mp=protocol supplementary concept, rare disease supplementary concept, title, original title, abstract, name of substance word, subject heading word, unique identifier] (796651)

2 (breast and (tumour or tumor or carcinoma)).mp. [mp=protocol supplementary concept, rare disease supplementary concept, title, original title, abstract, name of substance word, subject heading word, unique identifier] (108754)

3 breast cancer.mp. or Breast Neoplasms/ (198477)

4 advanced.mp. (192337)

5 2 or 3 (212370)

6 1 and 5 (21473)

7 6 not 4 (20047)

8 limit 7 to (yr="2000 -Current" and female) (10377)

9 trial.mp. or Randomized Controlled Trial/ (685227)

10 8 and 9 (1723)

Recurrence search

Database: Ovid MEDLINE(R) <1948 to January Week 3 2011>, Ovid MEDLINE(R) Daily Update <January 28, 2011>, Ovid MEDLINE(R) In-Process & Other Non-Indexed Citations <January 28, 2011>

Search Strategy:

----

1 (follow up or follow-up or surveillance).mp. [mp=protocol supplementary concept, rare disease supplementary concept, title, original title, abstract, name of substance word, subject heading word, unique identifier] (796651)

2 (breast and (tumour or tumor or carcinoma)).mp. [mp=protocol supplementary concept, rare disease supplementary concept, title, original title, abstract, name of substance word, subject heading word, unique identifier] (108754)

3 breast cancer.mp. or Breast Neoplasms/ (198477)

4 advanced.mp. (192337)

5 2 or 3 (212370)

6 1 and 5 (21473)

7 6 not 4 (20047)

8 limit 7 to (yr="2000 -Current" and female) (10377)

9 Mammography/ or mammograph*.mp. (25135)

10 8 and 9 (1602)

Population search

Database: Ovid MEDLINE(R) <1948 to January Week 3 2011>, Ovid MEDLINE(R) Daily Update <January 28, 2011>, Ovid MEDLINE(R) In-Process & Other Non-Indexed Citations <January 28, 2011>

Search Strategy:

----

1 (follow up or follow-up or surveillance).mp. [mp=protocol supplementary concept, rare disease supplementary concept, title, original title, abstract, name of substance word, subject heading word, unique identifier] (796651)

2 (breast and (tumour or tumor or carcinoma)).mp. [mp=protocol supplementary concept, rare disease supplementary concept, title, original title, abstract, name of substance word, subject heading word, unique identifier] (108754)

3 breast cancer.mp. or Breast Neoplasms/ (198477)

4 advanced.mp. (192337)

5 2 or 3 (212370)

6 1 and 5 (21473)

7 6 not 4 (20047)

8 limit 7 to (yr="2000 -Current" and female) (10377)

9 Population Groups/ or Population Surveillance/ or Rural Population/ or Urban Population/ or Suburban Population/ or population.mp. (770114)

10 8 and 9 (1849)
