# Supplementary material for: Options for early breast cancer follow-up in primary and secondary care - a systematic review
Source: BMC Cancer. 2012 Jun 13;12:238. doi: 10.1186/1471-2407-12-238 (PMC3502561; doi:10.1186/1471-2407-12-238)
Supplement: Additional file 3 — Inclusion criteria. [file 1471-2407-12-238-S3.pdf]

ADDITIONAL FILE 3 - Inclusion Criteria

1. Incidence of second breast cancer over time

Table 1. Incidence of second breast cancer over time – population studies

Included

Population based longitudinal studies from large breast cancer registries which had a high ascertainment and also reported incidence rate ratios for contralateral breast cancer.

Excluded

1. Patients with advanced cancer (stage4+)
2. Small studies reporting only one case series.
3. Case reports

2. Method of detection of local recurrence and second ipsi and contra-lateral breast cancer

Table 2. Incidence and method of detection of local recurrence and survival – reviews

Included

1. Cancer stage specified (1-3) with no metastases
2. Age specified
3. Cohorts followed up for a minimum of 5 years

Excluded

1. Cross-sectional studies
2. Patients with advanced cancer (stage4+)

Table 3. Method of detection of local recurrence and survival – cohort studies with report of surveillance mammography published after year 2000

Included

1. Cancer stage specified (1-3) with no metastases
2. Age specified
3. Prospective case series followed up as a cohort from diagnosis – any exclusions/losses specified and less than 30%
4. Cohorts followed up for a minimum of ten years
5. Surveillance mammography specified
6. Type of surgery specified
7. Survival measured from diagnosis of original cancer

Excluded

1. Patients from randomised controlled trials and other trials where consent was required
2. Studies with patients enrolled before 1980
3. Cross-sectional studies
4. Patients with advanced cancer (stage4+)

3. Evidence of the benefits of hospital or alternative setting follow-up for survival and well-being level 1 – 4 evidence

Table 4. Randomised controlled trials of breast cancer follow-up in hospital or alternative settings which include recurrence or survival as outcome – level 1 evidence

Included

1. RCTs
2. Outcome: local recurrence, contralateral disease, distant metastases or death
3. Follow up minimum 12 months

Excluded

1. Studies including women with stage 4 cancer
2. Studies of special populations
3. Chemotherapy or radiotherapy trials
4. Other articles not relevant eg. Studies of surgical procedures, diagnostic procedures, mammography utilization.

Table 5 Randomised controlled trials of breast cancer follow-up in hospital or alternative settings with acceptability, well-being, access to medical care as outcomes – level 1 evidence

Included

RCTs

1. Measures of Anxiety , depression, psychological morbidity, breast cancer related quality of life or patient satisfaction as outcomes
2. Randomisation procedure defined
3. Either stage 1 or stage 1 to 3 breast cancer
4. No signs of recurrence at recruitment
5. Population defined

Excluded

1. Studies including women with stage 4 cancer
2. No other exclusion criteria were used but limitations of the individual studies are discussed.

Table 6 Observational studies or audits of breast cancer follow-up in hospital or alternative settings which include acceptability, well-being or access to medical care as outcomes. – Level 2-4 evidence

Included

1. Measures of Anxiety , depression, psychological morbidity, breast cancer related quality of life or patient satisfaction as outcomes
2. Either stage 1 or stage 1 to 3 breast cancer
3. No signs of recurrence at recruitment

Excluded

1. Studies including women with stage 4 cancer
2. No other exclusion criteria were used but limitations of the individual studies are discussed.
